# Circular RNA RBM33 contributes to extracellular matrix degradation *via* miR-4268/EPHB2 axis in abdominal aortic aneurysm

**DOI:** 10.7717/peerj.12232

**Published:** 2021-11-16

**Authors:** Shizhi Wang, Qingwen Yuan, Wenpeng Zhao, Weimin Zhou

**Affiliations:** Department of Vascular Surgery, The Second Affiliated Hospital of Nanchang University, Nanchang, China

**Keywords:** Abdominal aortic aneurysm, Circular RNAs, circRBM33, Extracellular matrix degradation, EPH receptor B2, Matrix metalloproteinase-2, Tissue inhibitor of matrix metalloproteinases-1

## Abstract

**Background:**

Abdominal aortic aneurysm (AAA) is a complex vascular disease involving expansion of the abdominal aorta. Extracellular matrix (ECM) degradation is crucial to AAA pathogenesis, however, the specific molecular mechanism remains unclear. This study aimed to investigate differentially expressed circular RNAs (DEcircRNAs) involved in ECM degradation of AAA.

**Methods:**

Transcriptome sequencing was used to analyze the DEcircRNAs between the AAA tissues and normal tissues. The expression of circRNAs in tissues and cells was validated using quantitative reverse transcription PCR (RT-qPCR). Overexpression of circRNAs in vascular smooth muscle cells (VSMCs) treated with angiotensin II (Ang II) was employed to explore its effect on ECM degradation of AAA. Bioinformatic technology, luciferase reporter gene assay, RT-qPCR, and rescue experiment were employed to evaluate the regulatory mechanism of circRNA.

**Results:**

We identified 65 DEcircRNAs in AAA tissues compared with normal abdominal aortic tissues, including 30 up-regulated and 35 down-regulated circRNAs, which were mainly involved in inflammation and ECM-related functions and pathways. Moreover, circRBM33 was significantly increased in AAA tissues and Ang II-induced VSMCs compared with control samples. Overexpression of circRBM33 increased the expression of ECM-related molecule matrix metalloproteinase-2 and reduced the tissue inhibitor of matrix metalloproteinases-1 expression. Mechanistically, miR-4268 targeted binding to circRBM33 and inhibited the luciferase activity of circRBM33. Overexpression of circRBM33 induced the expression of EPH receptor B2 (EPHB2), and this effect was countered by miR-4268 mimics.

**Conclusions:**

Overall, our data suggest that circRBM33 might be involved in AAA progression by regulating ECM degradation *via* the miR-4268/EPHB2 axis.

## Introduction

Abdominal aortic aneurysm (AAA) is a common arterial aneurysm characterized by a localized, permanent dilatation of the aorta (Wang et al., 2014). The prevalence of AAA varies across approximately 1–2% of the population in men over the age of 65 years and 0.5% in women over the age of 70 years (Sakalihasan et al., 2018). AAA in most cases is asymptomatic, however, as the diameter of the aorta increases, the risk of AAA rupture increases, resulting in fatal bleeding (Xue et al., 2019). Currently, AAA treatment has chiefly been drug therapy and surgical intervention. A large proportion of AAA patients are older, and some has been accompanied by cardiovascular disease, including obesity, high lipid levels and high blood pressure. Accordingly, the risk of surgical treatment is significant, and clinical drugs are not effective in reducing the growth rate of tiny aneurysms (Cao et al., 2011; Ouriel et al., 2010). As a result, it is imperative to dig into the underlying mechanism in AAA in order to develop effective treatment strategies for AAA patients.

It has been reported that extracellular matrix (ECM) degradation, loss of arterial wall integrity, infiltration of inflammatory cells, and apoptosis of vascular smooth muscle cells (VSMCs) contribute to the pathogenesis of AAA (Quintana & Taylor, 2019; Zhang et al., 2019). The apoptosis and depletion of VSMCs are involved in AAA pathogenesis *via* removing a cell population that facilitates connective tissue repair (Sachdeva et al., 2017). The infiltrates of inflammatory cells occurred in both the media and adventitia, which is related with aneurysm diameter (Horimatsu et al., 2020). These cells generate proteolytic enzymes such as matrix metalloproteinases (MMPs), involving the ECM degradation in aortic walls. The ECM, as a highly dynamic structure, is a composite of macromolecules secreted by cells into the extracellular space (Hsu et al., 2019). ECM has been implicated in the regulation of various tissue development and cell physiology (Garde & Sherwood, 2021). Under physiological conditions, ECM plays an important role in the regulation of organogenesis, tissue differentiation and remodeling (Carreca et al., 2020; Peng et al., 2021). While under pathophysiological conditions, the change in the ECM composition is often closely associated with tumor infiltrating and the progression of tissue fibrosis (Jensen et al., 2018; Qorri et al., 2018). Previous studies have demonstrated that ECM remodeling is essential to AAA pathogenesis (Qin et al., 2012). Human AAA proteomic studies verified the change in expression and degradation of the ECM constituents, including collagen type XII, thrombospondin 2, aortic medial amyloid, muscle protein, and fibronectin (Didangelos et al., 2011). The MMPs and their endogenous inhibitors, tissue inhibitors of MMPs (TIMPs) are responsible for ECM metabolism (Li et al., 2020a). The increase of MMPs expression and the decrease of TIMPs expression facilitate ECM disruption and VSMC depletion, resulting in the progression and rupture of AAA (Zhang et al., 2019). In calcium chloride induced AAA mice model, MMP-14 derived from macrophages causes degradation of ECM, which promotes the formation of AAA (Xiong et al., 2009). However, intrinsic molecular mechanisms of ECM degradation in the AAA remain largely unknown.

As a class of non-coding RNAs, circular RNAs (circRNAs) can govern gene transcription at the transcriptional level and carry out various biological functions (Zheng et al., 2019). The functions of circRNAs in AAA have been reported. Microarray analysis of four paired aortic samples determines 411 differentially expressed circRNAs (DEcircRNAs) and reveals novel circRNAs potentially involved in the AAA pathogenesis (Zhou et al., 2020). The circCCDC66 expression in VSMCs treated with Angiotensin II (AngII) is upregulated, and the circCCDC66/miR-342-3p/CCDC66 axis regulates the proliferation and apoptosis of VSMCs (Yang et al., 2020b). MiR-181b in AAA negatively modulates the expression of macrophage tissue inhibitor of metalloproteinase-3 (TIMP3) (Di Gregoli et al., 2017), and hsa-circ-0005360 is predicated to bind to miR-181b and may regulate the progression of AAA (Zhou et al., 2020). In human AAA tissues, up-regulated hsa-circ-000595 binds to miR-19a to promote VSMCs apoptosis (Zheng et al., 2015). These studies demonstrate that circRNAs mediate progression of AAA, however, the expression pattern of circRNAs in AAA by high throughput sequencing has not been reported.

In this study, we focus on the circRNA expression patterns in AAA and the molecular mechanisms underlying ECM degradation. High throughput sequencing in AAA tissues and normal abdominal aortic tissues was performed to explore the expression profile and DEcircRNAs. The expression of circRBM33 was confirmed in AAA tissues and cells by quantitative reverse transcription PCR (RT-qPCR). Overexpression of circRBM33 in VSMCs was constructed to investigate the regulatory effects and molecular mechanisms of circRBM33 on ECM degradation, exploring novel therapeutic targets for AAA.

## Materials & methods

### Tissue samples

A total of 23 AAA tissues were collected from the AAA patients (age 48–65, mean age 56.3, 12 male, 11 female) receiving surgical procedures at the Second Affiliated Hospital of Nanchang University. The inclusion criteria were as follows: (a) with confirmed AAA diagnosis; (b) no radiotherapy and chemotherapy prior to surgery; (c) available clinical information. Patients would be excluded if suffered from other tumors and serious diseases and their clinical data were incomplete. Normal abdominal aortic tissues were obtained from age- and gender-matched organ donors without aortic diseases. All the patients were informed and signed the informed consent. This study was approved by the Ethics Committee of the Second Affiliated Hospital of Nanchang University (Ethical Application Ref: Study Clinical Review (2020) No. 044). Tissue samples were sliced into small segments in liquid nitrogen, and further stored at −80 °C.

### RNA extraction, library construction and sequencing

Total RNA was extracted from AAA tissues (*N* = 3) and normal abdominal aortic tissues (*N* = 3) with a Trizol RNA extraction reagent (Sigma–Aldrich, Shanghai, China). The quantity and purity of RNA were detected by the NanoDrop spectrophotometer (Thermo Fisher Scientific Inc., Shanghai, China). The RNA was electrophoresed on 1.2% agarose gel to analyze the integrity. For RNA-seq library construction, 1 μg of total RNA was used. The RNA libraries were constructed using the Total RNA-seq (HMR) Library Prep Kit (Azyme Biotech Co., Ltd., Nanjing, China). In brief, ribosomal RNA was removed with rRNA probes and RNase H, RNA was fragmented to ∼300 bp by metal ion, reverse transcribed into cDNA, and connected with adapters. The fragments were sorted by DNA Clean Beads (Beckman, USA), and subjected to PCR amplification and purification and libraries were validated using Agilent 2200 (Agilent, Santa Clara, CA, USA). Illumina platform (Illumina, Inc., CA, USA) was used for RNA sequencing.

### RT-qPCR

Total RNA from tissues (*N* = 3) and cells (*N* = 3) were extracted using TRIzol reagent (Invitrogen, CA, USA) according to the manufacture’ introduction. After the detection of RNA quality, purity, and content, 1 μg of RNA was reverse-transcribed into cDNA with a PrimeScript™ RT reagent kit (K1622; Thermo Fisher Scientific Inc., Shanghai, China). The PCR was performed with the SYBR Green PCR kit (Roche, Basel, Switzerland) on an ABI Q6 Real-time PCR system (Applied Biosystems Inc., Foster City, CA, USA). The reaction conditions were: 10 min of pre-denaturation at 95 °C, 45 cycles of denaturation at 95 °C for 15 s, and annealing at 60 °C for 60 s. The internal references in RT-qPCR were GAPDH and U6. Three independent experiments were conducted. The relative expression of each factor was evaluated using the 2^−ΔΔCt^ method. The RT primer and special PCR primers were listed in Table S1.

### Western Blot

Protein was extracted from cells with ice-cold RIPA buffer (Cell Signaling Technology, Danvers, MA, USA) containing a cocktail of protease inhibitors. Protein concentration was measured by BCA assay kit (Thermo Fisher Scientific Inc., Shanghai, China). Protein samples (50 µg) were separated in 10% SDS-PAGE gels and transferred to polyvinylidene difluoride (PVDF) membranes (Sigma-Aldrich, Shanghai, China). Membranes were blocked in 5% skim milk powder for 2 h at room temperature and incubated overnight at 4 °C with primary antibodies. The primary antibodies applied are as follows: rabbit anti-human tissue inhibitor of matrix metalloproteinases-1 (TIMP-1; ab109125, 1:1000, abcam, Shanghai, China), rabbit anti-human EPH receptor B2 (EPHB2; ab216629, 1:2000, abcam, Shanghai, China), and rabbit anti-human GAPDH (ab9485, 1:2000, abcam, Shanghai, China). The membranes were then washed three times with TBST for 10 min each and incubated with goat anti-rabbit IgG-HRP antibody (ab205718, 1:20,000, abcam, Shanghai, China). After three washes with TBST, membranes were subjected to ECL chemiluminescence reagent (Thermo Fisher Scientific Inc., Shanghai, China). Images were obtained using a ChemiDoc Gel Imaging system (Thermo Fisher Scientific Inc., Shanghai, China), and the optical density was quantified with Quantity One image analysis software (Bio-rad, Hercules, CA, USA). Experiments were performed in triplicate.

### Luciferase reporter assay

The circRBM33 wild-type (WT, containing miR-4268 binding sites) and mutant 3′-UTR fragments (Mut, mutant miR-4268 binding sites) were cloned into a psicheck-2 vector. The 293T cells were seeded into 48-well plates and cultured for 24 h. Then, the reporter gene vectors were co-transfected with miR-4268 mimics or NC mimics into cells using Lipofectamine 2000 (Invitrogen, Carlsbad, CA, USA). After 48 h transfection, the luciferase activity in each group was examined by a Dual-Luciferase Assay Kit (Promega, Madison, WI, USA). The experiment was repeated thrice.

### Statistical analysis

All of these experiments were repeated three times. Statistical analyses were assessed with the SPSS v.21.0 software (IBM Corp, Armonk, NY, USA). Data were expressed as mean ± SD. The comparisons of means among the two groups were evaluated by Student’s t-test. One-way ANOVAs were performed for multiple comparisons, and the Tukey-HSD *post hoc* test was used. For all tests, *p* < 0.05 was considered to be statistically significant.

## Results

### Analysis of DEcircRNAs

To investigate the expression profiles of circRNAs implicated in AAA, RNA sequencing was performed on samples from AAA tissues and normal abdominal aortic tissues. After filtering low-quality and duplicated reads (filtration rate > 89%), approximately 9.1–11.2 million of clean reads were obtained in each sample (Table S3). The clean reads were then aligned onto the human (GRCh38) reference genome, the alignment rates of clean reads ranged from 89% to 96%, indicating that the sequencing data was reliable. Approximately 0.9–1.8 million junction reads were selected for circRNA prediction and quantification, and 5621 (AAA-1), 7190 (AAA-2), 5547 (AAA-3), 7402 (Con-1), 8233 (Con-2), 8480 (Con-3) were generated in six tissue samples, respectively. After filtering of lowly-expressed circRNAs, 1,081, and 1,372 were respectively obtained in the AAA group and control group, and 200 circRNAs were co-expressed in these two groups (Fig. S1).

**Figure 1:**
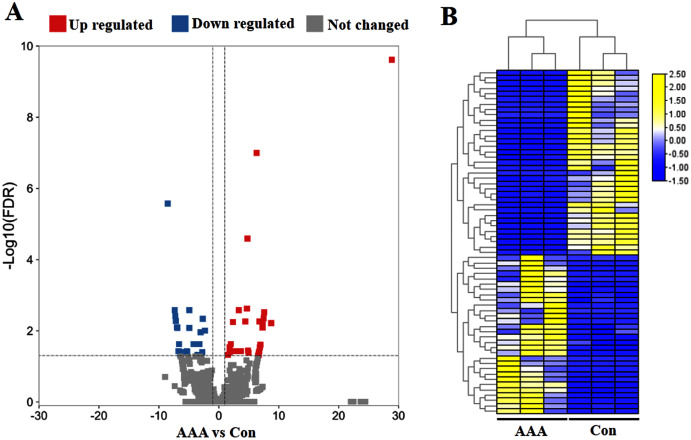
Analysis of differentially expressed circRNAs (DEcircRNAs). (A) Volcano plots of circRNAs in abdominal aortic aneurysm (AAA) tissues from patients (*N* = 3) and abdominal aortic tissues from healthy subjects (*N* = 3) (normal tissues). The red and blue circle represent up- and down-regulated DEcircRNAs (|log2 fold change| ≥ 1 and *p* value < 0.05), respectively. (B) Heatmap of DEcircRNAs in AAA tissues and normal tissues. The high to low expression levels were represented as red to green. AAA: AAA group. Con: control group (normal tissues).

To identify circRNA expression signatures in AAA, the DEcircRNAs were identified by the selection criteria of FDR <0.05, log2 fold change >1 or <−1. In total, 65 significant DEcircRNAs were recognized, including 30 up-regulated DEcircRNAs and 35 down-regulated DEcircRNAs in the AAA tissues compared with the normal control group (Fig. 1A). The six samples were clustered closely into two groups, namely the AAA group and the control group, indicating a difference of circRNA expression between the AAA group and the control group (Fig. 1B).

### Functional and pathway analysis of DEcircRNAs

**Figure 2:**
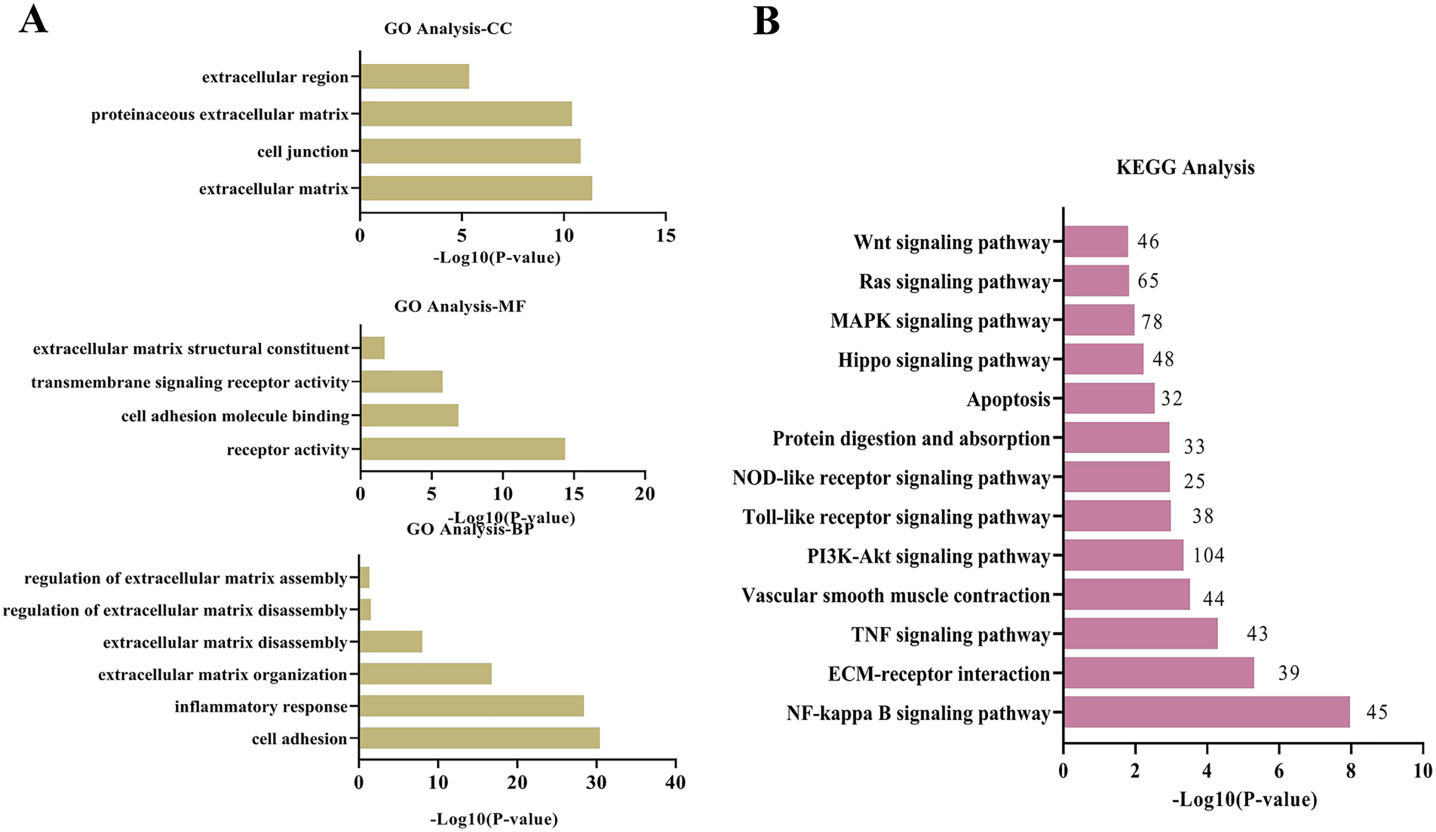
Functional and pathway analysis of DEcircRNAs. (A) GO enrichment analysis based on the target genes of DEcircRNAs. (B) The top 20 enriched pathways of target genes of DEcircRNAs. X-axis: −log10 (*P*-value); Y-axis: GO terms or pathways.

To explore wthether the DEcircRNAs were associated with ECM degradation, the GO and KEGG analysis was performed. The target genes modulated by DEcircRNAs were enriched in biological process (BP) of “cell adhesion”, “inflammatory response”, “ECM organization”, “ECM disassembly”, “regulation of ECM disassembly”, and “regulation of ECM assembly”; the top terms in cellular component (CC) were “ECM” “cell junction”, “proteinaceous ECM”, and “extracellular region”; these DEcircRNAs were found to be involved in molecular function (MF) of “receptor activity”, “cell adhesion molecule binding”, “transmembrane signaling receptor activity”, and “ECM structural constituent”(Fig. 2A). KEGG enrichment analysis for DEcircRNAs were assigned into ECM-related pathways, including TNF signaling pathway (Mei et al., 2019), NF-κB signaling pathway (Guo et al., 2021), ECM-receptor interaction (Lu et al., 2011), and PI3K-Akt signaling pathway (Gao et al., 2020) (Fig. 2B).

### circRBM33 is highly expressed in AAA

**Figure 3:**
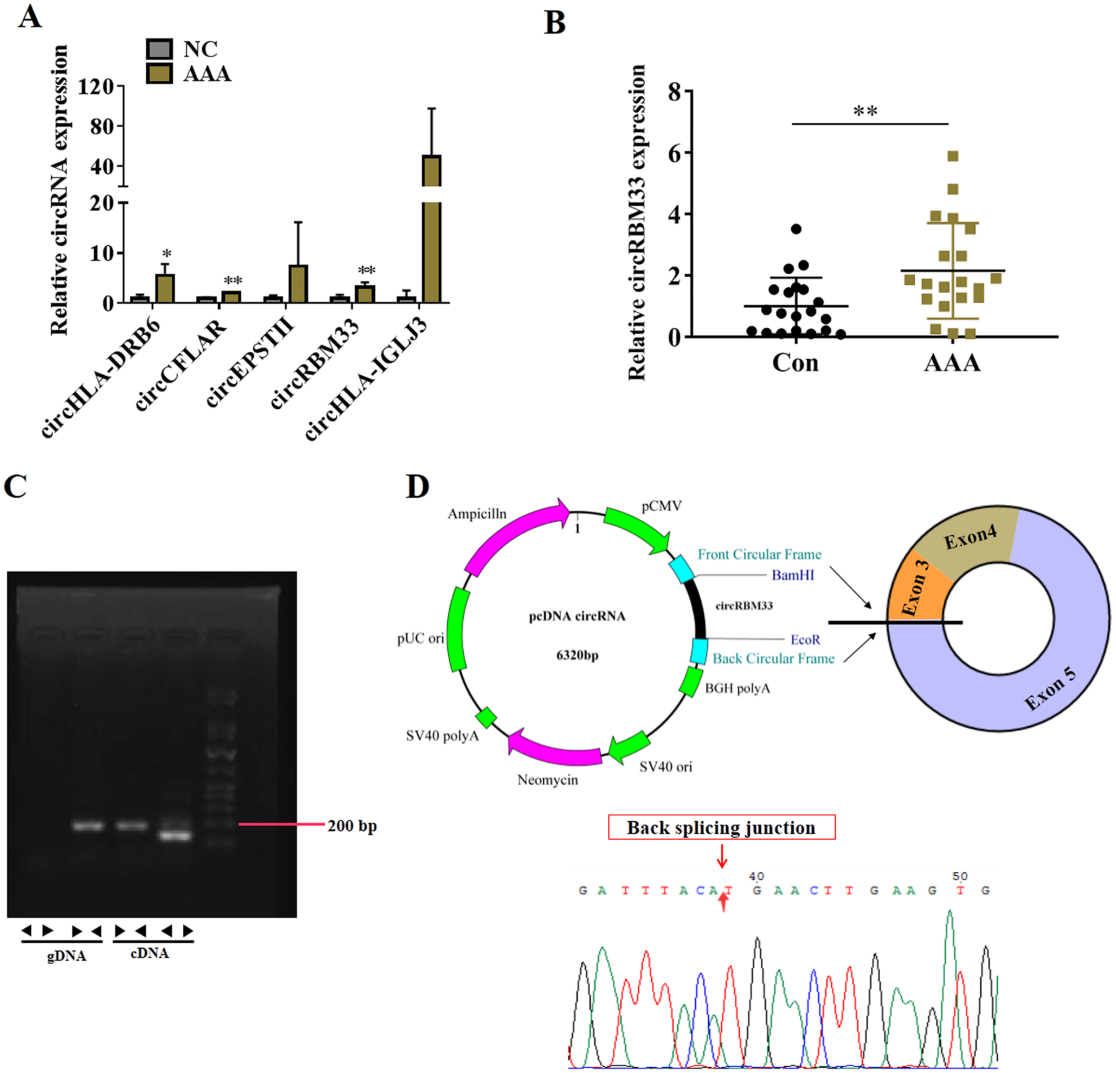
circRBM33 is highly expressed in AAA. (A) RT-qPCR analysis of five candidate DEcircRNAs in AAA tissues (*N* = 3) and normal tissues (*N* = 3). (B) The expression of circRBM33 in AAA tissues (*N* = 20) and normal tissues (*N* = 20) measured by RT-qPCR. (C) Validation of circRBM33in cDNA using divergent primers. (D) Schematic presentation of circRBM33 circularization. Data are expressed as mean ± SD. *p < 0.05, **p < 0.01.

To seek for key circRNAs in the progression of AAA, five circRNAs (circHLA-DRB6, circCFLAR, circEPSTII, circRBM33, circHLA-IGLJ3) with higher expressed abundance, more fold change and higher significance were selected for RT-qPCR examination in six sequencing samples (Fig. 3A). Obviously, the expression of circHLA-DRB6, circCFLAR and circRBM33 had statistical differences between the AAA samples and normal samples. Two studies have showed that circRBM33 (chr7_155680908_155672867_+8041-RBM33, generated from human gene RBM33, termed circRBM33) is the pro-oncogenic factor in cervical cancer and gastric cancer (Wang et al., 2020; Ding, Yuan & Gu, 2021). Moreover, the expression of circRBM33 in AAA tissues was significantly up-regulated compared with that in the normal abdominal aortic tissues (Fig. 3B). Divergent and convergent primers were designed to amplify circRBM33 in gDNA and cDNA of AAA tissues, and found only the divergent primers amplified circRBM33 from cDNA, whereas, no amplification product was visualized from gDNA (Fig. 3C). The PCR products were purified and subjected to Sanger sequencing to detect the back-splicing exon junction regions of circRBM33. The sequencing data showed that circRBM33 was generated from exon 3 and exon 5 of RBM33 gene by back-splicing (Fig. 3D).

### Overexpression of circRBM33 induces the ECM degradation

**Figure 4:**
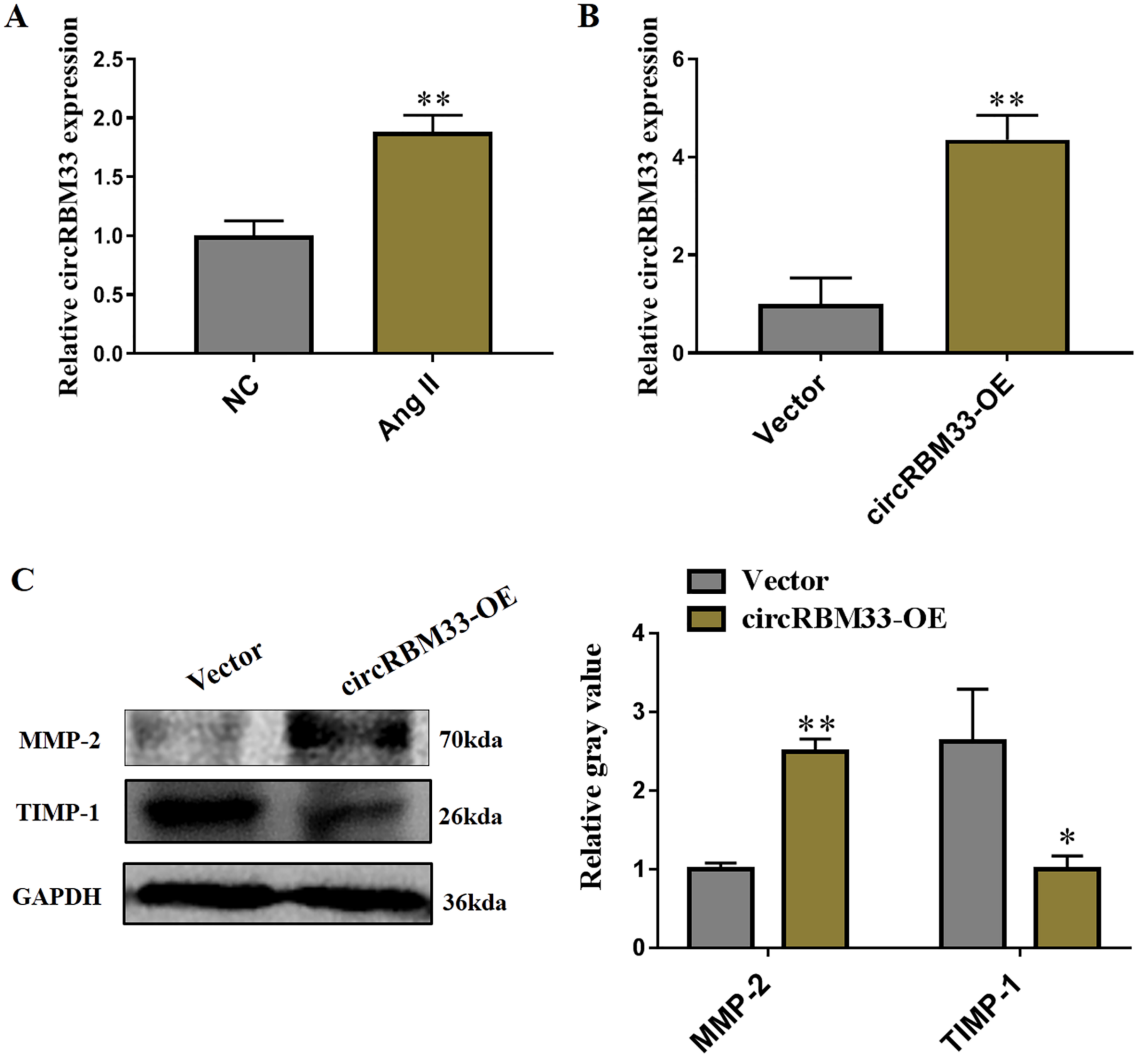
Overexpression of circRBM33 induces the extracellular matrix (ECM) degradation. (A) The expression of circRBM33 in Ang II-induced vascular smooth muscle cells (VSMCs) was detected by RT-qPCR. (B) The expression of circRBM33 in Ang II-induced VSMCs with circRBM33 overexpression or control vector plasmids transfection was measured by RT-qPCR. (C) MMP-2, TIMP-1 expression in Ang II-treated VSMCs in each group was detected by western blot. Data are expressed as mean ± SD. *p < 0.05, **p < 0.01. Experiments were run in triplicate.

Subsequently, we evaluated the roles of circRBM33 in AAA progression. AAA model was mimicked in human VSMCs *via* inducing by Ang II to measure the expression. Compared with the blank control group, the circRBM33 expression in Ang II-induced group was significantly increased (Fig. 4A). Overexpression of circRBM33 in VSMCs was mediated by circRBM33 overexpression vector. RT-qPCR analysis found that transfection with circRBM33 overexpression vector resulted in a significant up-regulation of circRBM33 in VSMCs (*P* < 0.01, Fig. 4B). Numerous enzymes have been reported to be involved in regulating ECM degradation, including MMP-2 and TIMP-1 (Yuan & Wu, 2018; Guo et al., 2019). MMP-2 is well documented to promote the degradation of ECM (Miyakawa et al., 2019), and TIMP-1 attenuates the ECM degradation by inhibiting nearby MMPs (Nordgaard et al., 2019). Thus, we determined MMP-2 and TIMP-1 expression in the circRBM33 overexpressing VSMCs by western blot to evaluate the effect of circRBM33 on ECM degradation. As expected, the level of MMP-2 in VSMCs with circRBM33 overexpression was significantly increased relative to the control cells, and the TIMP-1 expression was reduced in VSMCs with circRBM33 overexpression (Fig. 4C).

### circRBM33 mediates the ECM degradation by directly binding to miR-4268

**Figure 5:**
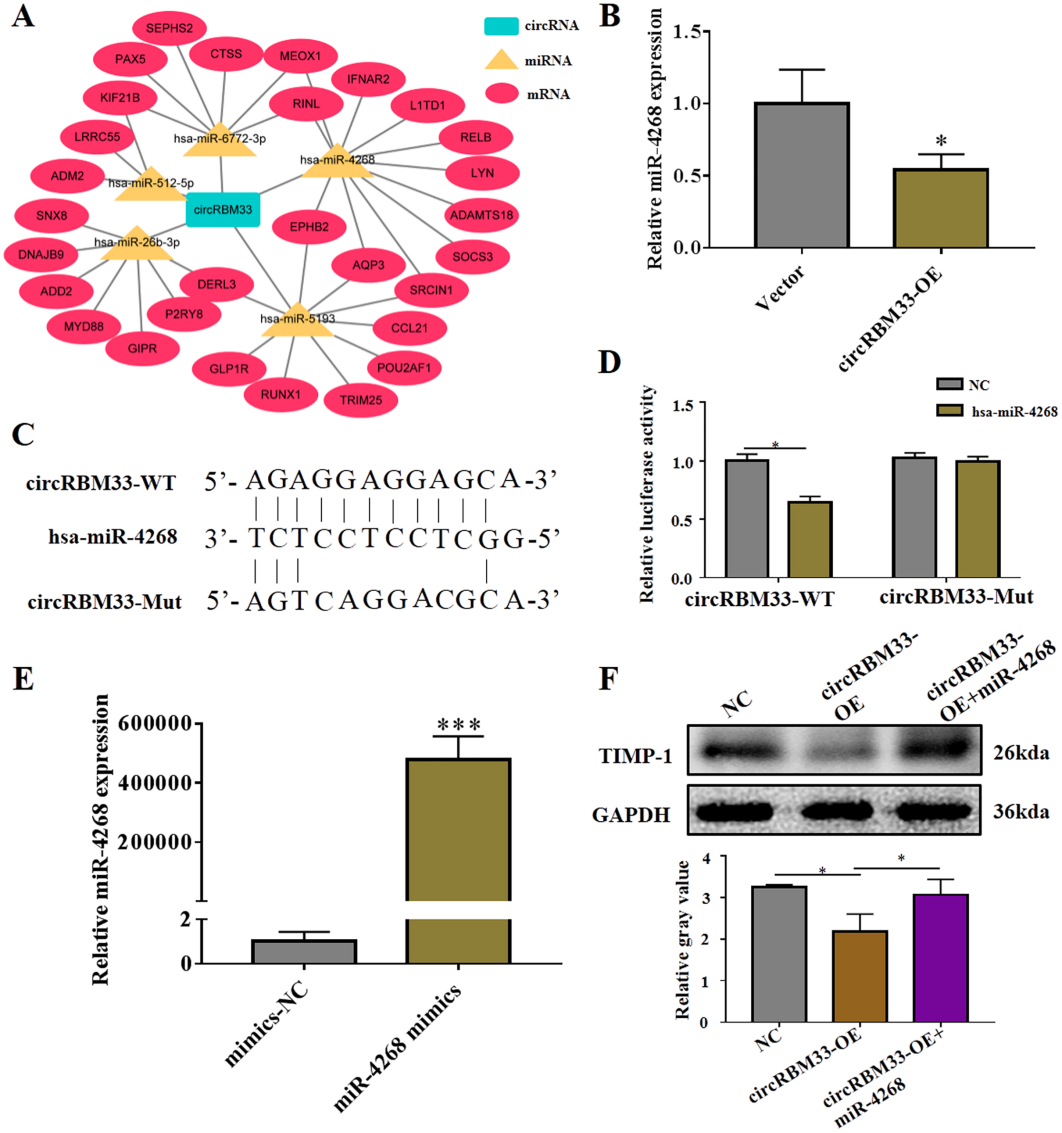
circRBM33 mediates the ECM degradation by directly binding to miR-4268. (A) The circRNA-miRNA-mRNA network for circRBM33. (B) The expression of miR-4628 in circRBM33- overexpressed VSMCs was examined by RT-qPCR. (C) Bindings sites among circRBM33-Mut, circRBM33-WT and miR-4628. (D) Luciferase activity of circRBM33 in VSMCs transfected with miR-4628 mimics. (E) The expression of miR-4628 in VSMCs transfected with miR-4268 mimics or control mimics was examined by RT-qPCR. (F) TIMP-1 expression was detected by western blot. Data are expressed as mean ± SD (*N* = 3). *p < 0.05, **p < 0.01. ***p < 0.001.

To explore the mechanism of circRBM33 in ECM degradation and AAA progression, we selected the potential targets of circRBM33 and created a circRNA-miRNA-mRNA interaction network (Fig. 5A). The miR-4268, which targeted several oncogenic genes (L1TD1, LYN, SOCS3, SRCIN1, AQP3, EPHB2), was detected in the VSMCs. We found that the overexpression of circRBM33 caused a significant reduction in the miR-4268 expression (Fig. 5B). Furthermore, the binding sites of miR-4268 and circRBM33 were predicted by bioinformatics analysis, and the binding sites was exhibited in Fig. 5C. For luciferase reporter gene assay, the mutant luciferase reporters and the WT luciferase reporters were constructed. Luciferase activity assay showed that miR-4628 mimic significantly decreased the luciferase activity of the WT group but not the Mut group (Fig. 5D), suggesting the direct binding of circRBM33 with miR-4628. The miR-4268 mimics was transfected into VSMCs, and induced the miR-4268 expression in VSCMs as compared to the control cells (Fig. 5E). Moreover, the decreasing TIMP-1 expression in the VSMCs caused by circRBM33 overexpression was rescued by miR-4268 mimics (Fig. 5F), indicating that circRBM33 mediates the ECM degradation by directly binding to miR-4268 in AAA.

### circRBM33 regulates EPHB2 expression

**Figure 6:**
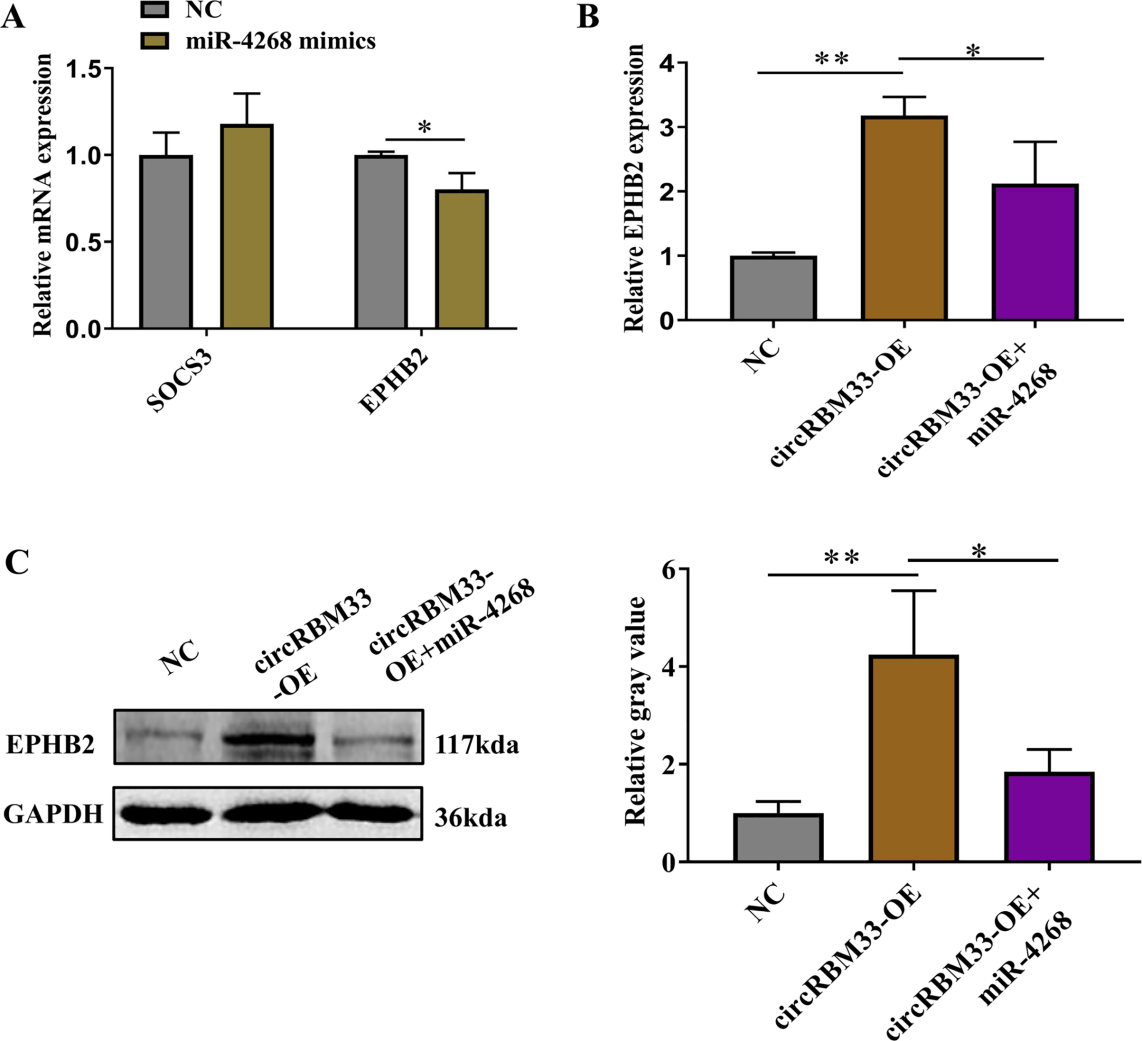
circRBM33 regulates EPHB2 expression. (A) SOCS3 and EPHB2 expression in VSMCs transfected with miR-4268 mimics or control mimics was examined by RT-qPCR. (B) The expression of EPHB2 in VSMCs was examined by RT-qPCR. (C) EPHB2 expression was detected by western blot. Data are expressed as mean ± SD (*N* = 3). *p < 0.05, **p < 0.01.

Previous studies on circRNAs have indicated that circRNAs can regulate genes by acting as miRNA sponges. Here, we screened two predicted target genes (SOCS3, EPHB2) of miR-4268 for further study. In addition, the expression of EPHB2 was significantly decreased, and that of SOCS3 had no significant change in VSMCs with miR-4268 overexpression (Fig. 6A). To verify whether circRBM33 exerts its role by sponging miR-4268, we carried out the rescue experiment. As shown in Fig. 6B and 6C, the mRNA and protein levels of EPHB2 were increased in the circRBM33 overexpressing VSMCs, and the increase was partly reversed by miR-4268 mimics.

## Discussion

It is reported that an increased replacement of ECM collagen without adequately matched collagen deposition is a critical factor for AAA rupture in the dissection (Jana et al., 2019; Adams et al., 2020). There are various underlying mechanisms implicated in the pathogenesis of AAA, including ECM degradation. However, the specific mechanism of ECM degradation in AAA remains largely unknown. Here, 65 DEcircRNAs were identified in AAA tissues using RNA sequencing, which were involved in immune response and ECM degradation. We found that circRBM33 was significantly up-regulated in AAA clinical samples as well as cell models. Furthermore, the overexpression of circRBM33 in VSMCs markedly induced the expression of ECM degradation-related gene MMP-2, and reduced the expression of TIMP1. Mechanistically, circRBM33 mediated the ECM degradation by regulating EPHB2 expression *via* hsa-miR-4268.

Following the development of sequencing technologies, more novel circRNAs has been dug out and proved to play an important role in tumor (Dai et al., 2019; Ma et al., 2019; Yang et al., 2020a). Multiple circRNAs were involved in the regulation of AAA. A recent study demonstrated that circCBFB modulated VSMC apoptosis and proliferation in AAA *via* the miR-28-5p/GRIA4/LYPD3 axis (Yue et al., 2020). circCCDC66 mediated the apoptosis and proliferation of VSMCs by inducing its host gene CCDC66, and thus facilitated AAA pathogenesis and progression (Yang et al., 2020b). Our study confirmed a series of DEcircRNAs between AAA tissues and normal tissues, which were involved in ECM-related pathways, such as NF-kappa B signaling pathway and cell adhesion molecules. Research showed that NF-κB is a transcription factor and modulates the inflammatory response in aneurysmal tissues (Tsai et al., 2018). In human and animal aneurysmal tissues, the NF-κB signaling pathway is activated (Liu et al., 2020). Inhibition of the NF-κB signaling pathway may result in the increase of MMP-2 and MMP-9 expression, regulate the ECM degradation, and ameliorate the AAA formation induced by AngII. Cell adhesion molecules are cell-membrane proteins that facilitate cell-cell contacts and adhesion with the ECM (Ayuk, Abrahamse & Houreld, 2016). The components of the ECM were closely associated with cell adhesion molecules (Hassani et al., 2019). Therefore, circRNAs play a key role in AAA progression by regulating ECM degradation.

In this study, we discovered that circRBM33 was significantly increased in AAA tissues and cell models compared with the normal group, and overexpression of circRBM33 enhanced the expression of MMP-2 and reduced TIMP-1 expression. In AAA, the apoptosis and ECM degradation of VSMCs were closely related to the increase of MMPs and the decrease of TIMP-1 (Cai et al., 2021). TIMPs are key regulators of ECM degradation and remodeling. Kurogi et al. (2015) discovered that up-regulation of TIMP-1 expression in the early stage of cerebral vasospasm may contribute to the recovery of ECM in the later stage of cerebral vasospasm. MMPs are key proteases in ECM degradation. MMPs could promote the development of AAA by degrading elastic and collagen fibers in aortic tissues, especially MMP-2 and MMP-9, which were considered as critical regulators in AAA progression (Li et al., 2020b). In hypoxic endothelial cells treated with tissue plasminogen activator, the release of MMP-2 contributes to ECM degradation (Song et al., 2016). Taken together, circ-RBM33 regulates AAA progression by regulating ECM degradation.

CircRNAs could exert biologic function by competitively binding to endogenous miRNAs to modulate gene transcription (Yu & Kuo, 2019; Jia et al., 2020). Here, we predicted the target genes of circRBM33 and constructed the circRNA-miRNA-mRNA network. We found that circRBM33 regulated the expression of EPHB2 by acting as miR-4268 sponge. EPHB2 belongs to a member of the receptor tyrosine kinase family, and has been shown to act as an oncogene in various cancers (Koh et al., 2020; Lucero et al., 2020). The EPHB2 expression in AAA was significantly increased (Sakamoto et al., 2012), and EPHB2 down-regulation reduced the mRNA expression levels of the MMPs (Goparaju et al., 2013). Moreover, the EPHB2 regulatory region existed multiple NF-κB binding sites (Pozniak, Darbinyan & Khalili, 2016), suggesting there was a tight connectivity among NF-κB and ECM. Functionally, we confirmed that the overexpression of circRBM33 in VSMCs significantly increased the mRNA and protein expression of EPHB2, and inhibited TIMP-1 expression, suggesting circ-RBM33 contributes to AAA progression by regulating ECM degradation. Taken together, circRBM33 may involve in AAA progression *via* the miR-4268/EPHB2 axis.

## Conclusions

This study confirmed that circRBM33 was up-regulated in AAA tissues and cell models. Overexpression of circRBM33 in VSMCs significantly induced ECM degradation and circRBM33 regulated the expression of EPHB2 by acting as miR-4268 sponge. Our results suggest that circRBM33 may be a potential diagnostic and therapeutic target for AAA.

## Supplemental Information

10.7717/peerj.12232/supp-1 Supplemental Information 1 Venn diagram of circRNAs in AAA tissues and adjacent normal tissues.

10.7717/peerj.12232/supp-2 Supplemental Information 2 Primer sequences for RT-qPCR.

10.7717/peerj.12232/supp-3 Supplemental Information 3 Primer sequences for circHLA-RBM33.

10.7717/peerj.12232/supp-4 Supplemental Information 4 Summary statistics of RNA sequencing data. AAA: AAA group. Con: control group (normal tissues).

10.7717/peerj.12232/supp-5 Supplemental Information 5 Raw data for RT-qPCR.

10.7717/peerj.12232/supp-6 Supplemental Information 6 The full length images of western blot results for Figs. 4C, 5F, 6C.

10.7717/peerj.12232/supp-7 Supplemental Information 7 Supplementary method.

## Additional Information and Declarations

### DNA Deposition

The following information was supplied regarding the deposition of DNA sequences: The RNA sequences described here are accessible *via* GenBank accession number PRJNA699407.

### Data Availability

The following information was supplied regarding data availability: The data is available at figshare: Zhou, Weimin (2021): Data Files. figshare. Dataset. https://doi.org/10.6084/m9.figshare.16441944.v3. The raw data for RT-qPCR and the western blots are available as Supplemental Files.
