# Supplementary figures and images for: Circular RNA RBM33 contributes to extracellular matrix degradation via miR-4268/EPHB2 axis in abdominal aortic aneurysm
Source: PeerJ. 2021 Nov 16;9:e12232. doi: 10.7717/peerj.12232 (PMC8603816; doi:10.7717/peerj.12232)

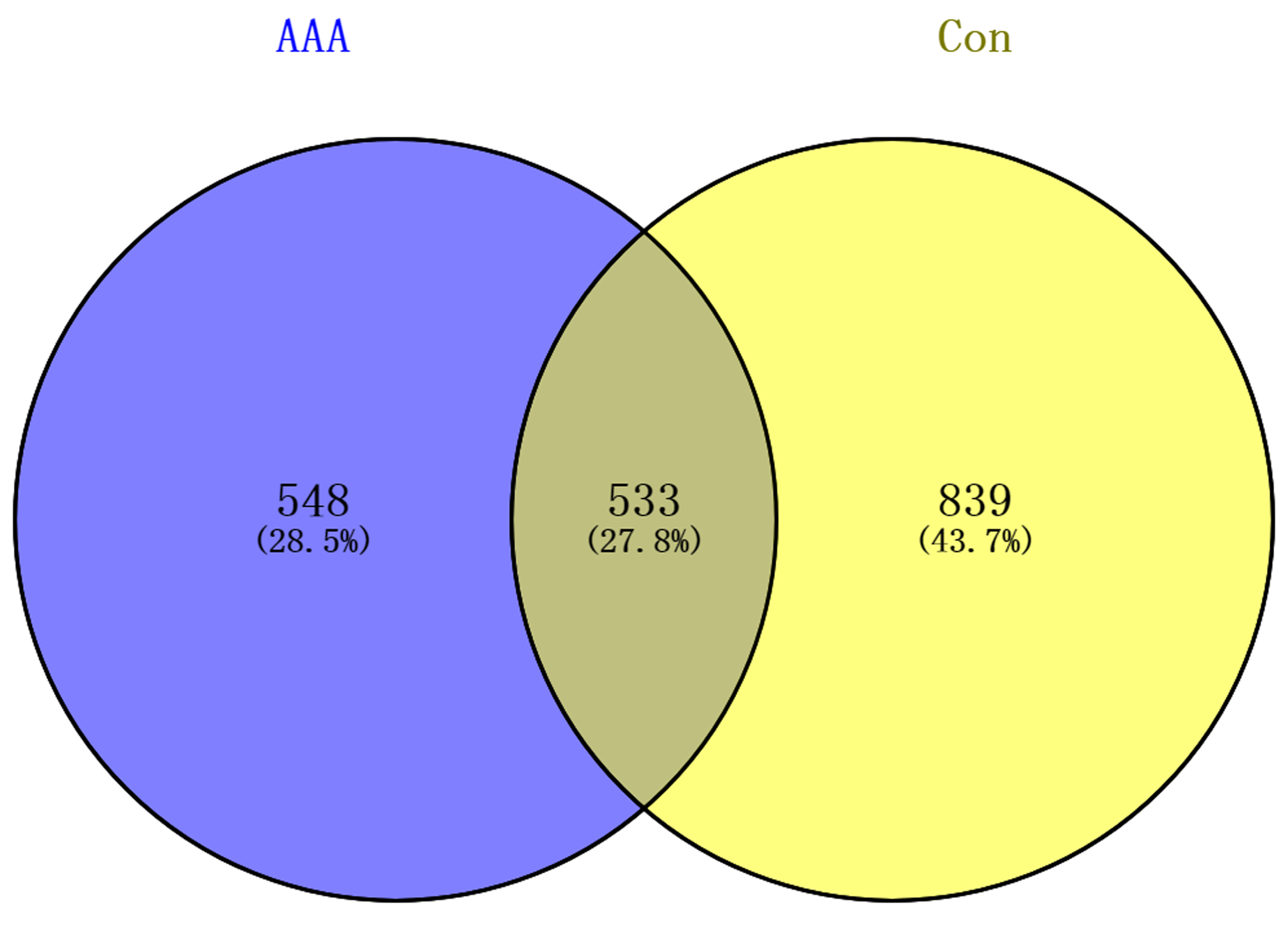

Supplement: Supplemental Information 1 [file peerj-09-12232-s001.png]
