# Supplementary material for: Circular RNA RBM33 contributes to extracellular matrix degradation via miR-4268/EPHB2 axis in abdominal aortic aneurysm
Source: PeerJ. 2021 Nov 16;9:e12232. doi: 10.7717/peerj.12232 (PMC8603816; doi:10.7717/peerj.12232)
Supplement: Supplemental Information 2 [file peerj-09-12232-s002.docx]

**Table S1: Primer sequences for RT-qPCR**

| **Primer** | **Sequences (5’ to 3’)** | **Tm/℃** |
| --- | --- | --- |
| GAPDH-F | AGAAGGCTGGGGCTCATT | 60 |
| GAPDH-R | TGCTAAGCAGTTGGTGGTG |  |
| circCFLAR-F | TGGCAATGAGACAGCTCCATA | 60 |
| circCFLAR-R | ACTGGTTCTTGTTGAGCGCC |  |
| circEPSTI1-F | GCATCAGCAATACACAAGTGCATAC | 60 |
| circEPSTI1-R | GCTCCTGCTCCGCAATTCTT |  |
| circHLA-DRB6-F | CTGCAGACACAACTACGGGG | 60 |
| circHLA-DRB6- R | GATGCAGGGGCTGGGTCTTT |  |
| circRBM33-F | CCAGAGGAGGAGCAGCTTTAC | 60 |
| circRBM33-R | AATCCGACTGATTCTTTTTGCCA |  |
| circHLA-IGLJ3-F | CACAGTGTTGGGTGTTCGG | 60 |
| circHLA-IGLJ3-R | CCCGGGTAGAAGTCACTTATGA |  |
| U6-F | CGATACAGAGAAGATTAGCATGGC | 60 |
| U6-R | AACGCTTCACGAATTTGCGT |  |
| hsa-miR-4268-F | GGCTCCTCCTCTCAGGAT | 60 |
| hsa-miR-4268-R | AGTGCGTGTCGTGGAGTCG |  |
| EPHB2-F | TGGACTCCACTACAGCGACT | 60 |
| EPHB2-R | TGCGGATCGTGTTCATGTTC |  |
