# Supplementary material for: Circular RNA RBM33 contributes to extracellular matrix degradation via miR-4268/EPHB2 axis in abdominal aortic aneurysm
Source: PeerJ. 2021 Nov 16;9:e12232. doi: 10.7717/peerj.12232 (PMC8603816; doi:10.7717/peerj.12232)
Supplement: Supplemental Information 3 [file peerj-09-12232-s003.docx]

**Table S2: Primer sequences for circHLA-RBM33**

| **Primer** | **Sequences** | **Product expected length/bp** | **Tm/℃** |
| --- | --- | --- | --- |
| divergent primer-F | CCAGAGGAGGAGCAGCTTTAC | 134 | 60 |
| divergent primer -R | AATCCGACTGATTCTTTTTGCCA |  |  |
| convergent primer-F | GCTACATCTGGCATGGTTACA | 182 | 60 |
| convergent primer-R | GCTTCGTGGCCTTCATACTG |  |  |
