# Supplementary material for: Circular RNA RBM33 contributes to extracellular matrix degradation via miR-4268/EPHB2 axis in abdominal aortic aneurysm
Source: PeerJ. 2021 Nov 16;9:e12232. doi: 10.7717/peerj.12232 (PMC8603816; doi:10.7717/peerj.12232)
Supplement: Supplemental Information 4 — AAA: AAA group. Con: control group (normal tissues). [file peerj-09-12232-s004.docx]

**Table S3: Summary statistics of RNA sequencing data**

| **Type** | **AAA-1** | **AAA-2** | **AAA-3** | **Con-1** | **Con-2** | **Con-3** |
| --- | --- | --- | --- | --- | --- | --- |
| Raw reads | 101802334 | 122742322 | 101828054 | 116986342 | 112631614 | 104671530 |
| Clean Reads | 91303870 | 111841652 | 92102492 | 105789744 | 103991464 | 95446548 |
| Filter Rate (%) | 89.7 | 91.1 | 90.4 | 90.4 | 92.3 | 91.2 |
| Mapped reads | 86912410 | 105264985 | 87712548 | 100592363 | 93473733 | 86816749 |
| Mapped Rate (%) | 95.2 | 94.1 | 95.2 | 95.1 | 89.9 | 91.0 |
| Junction Mapped reads | 17302260 | 11039382 | 9124364 | 12940955 | 14962600 | 14859893 |
| CirRNA number | 5621 | 7190 | 5547 | 7402 | 8233 | 8480 |

AAA: AAA group. Con: control group (normal tissues).
