# Supplementary material for: Circular RNA RBM33 contributes to extracellular matrix degradation via miR-4268/EPHB2 axis in abdominal aortic aneurysm
Source: PeerJ. 2021 Nov 16;9:e12232. doi: 10.7717/peerj.12232 (PMC8603816; doi:10.7717/peerj.12232)
Supplement: Supplemental Information 7 [file peerj-09-12232-s007.docx]

**1. Bioinformatics analysis**

The raw sequence data were evaluated using Fast-QC (<http://www.bioinformatics.babraham.ac.uk/projects/fastqc/>), and the clean reads were mapped to human reference genome (GRCh38) by HISAT2 software v2.1.0 (https://daehwankimlab.github.io/hisat2/). The reads that could not be mapped to the genome were collected for back-splicing junction mapping using ACFS2 (<https://github.com/arthuryxt/acfs>), which is available for circRNAs identification and quantification. The internationally recognized algorithm DESeq2.0 was adopted to identify the differentially expressed circRNAs (DEcircRNAs) with the threshold of false discovery rate (FDR) < 0.05 and |Log2 Fold Change| > 1. Subsequently, the volcano plots were used to visualize DEcircRNAs, and the hierarchical clustering heatmaps was plotted by using MEV software v4.9.0 ((http://mev.tm4.org). Predicting the target genes of DEcircRNAs was performed by miRanda (www.microrna.org) and RNAhybird (https://bibiserv.cebitec.uni-bielefeld.de/rnahybrid/). Kyoto Encyclopedia of Genes and Genomes (KEGG, http:\\www.genome.jp\kegg\) pathway and Gene Ontology terms (GO, http:\\www.geneontology.org\) annotation analysis were used to infer the functional roles of DEcircRNAs. Additionally, the miRNA-mRNAs interaction network analysis was constructed using Cytoscape software 3.6.1 (https://cytoscape.org/).

**2. Validation of circRBM33**

Total RNA from AAA tissues (N=20) and normal tissues (N=20) was extracted referring to the manufactur’ntroduction and was reverse-transcribed into cDNA. Genomic DNA (gDNA) was isolated by a genomic DNA extraction kit (Tiangen Biotech Co. Ltd., Beijing, China) and used as control. Based on the NCBI reference sequences of RBM33 (NCBI accession number: NC_000007.14) and circRBM33 sequence, divergent and convergent primers (Table S2) were designed to confirm the presence of circRBM33. The gDNA or cDNA (25 ng) were used as templates for PCR reaction. The PCR procedure was as follows: 95°C for 3 min; 35 cycles (94°C for 60 s, 60°C for 40s, 72°C for 30s), 72°C extension for 3 min. The PCR products were electrophoresed on 1.5% agarose gels, and sequenced by Sanger sequencing.

**3. Cell culture and management**

Human vascular smooth muscle cells (VSMCs) were purchased from Procell (Wuhan, China), and cultured in DMEM medium (Corning, NY, USA) supplemented with 10% fetal bovine serum (FBS, Gibco, USA). Cells were incubated at 37°C with 95% air and 5% CO_2_. Total 100 nM of Angiotensin II (Ang II; Sigma, Missouri, USA) was applied for treating VSMCs.

**4. Vectors construction and cell transfection**

To overexpress circRBM33, the full-length cDNA of circRBM33 fragment was cloned into the pcDNA3.1 (+) vector. Briefly, after cloning the stuffer fragments into the KpnI and BamHI sites of pcDNA3.1 vector, the circRBM33 vector was constructed by cloning the upstream and downstream intron fragments (300bp) of circRBM33 into the KpnI/Xho I and BamHI/EcoR I sites of the plasmid. The plasmid was transferred to *E. coli* Rosetta DH5α cells and amplified. Plasmid transfections into VSMCs were carried out by Lipofectamine 3000 (Invitrogen, Carlsbad, USA). The transfected cells were placed in a 37°C incubators for 24 h and RNA was extracted for RT-qPCR analysis.
